# Measles-Mumps-Rubella Vaccine and COVID-19 Relationship

**DOI:** 10.1128/mBio.01832-20

**Published:** 2020-09-22

**Authors:** Öner Özdemir

**Affiliations:** a Division of Allergy and Immunology, Department of Pediatrics, Research and Training Hospital of Sakarya University, Adapazarı, Sakarya, Turkey; Baylor College of Medicine; Harvard Medical School

## LETTER

I have read the article by Fidel and Noverr with great interest (1). As told in the article, could measles-mumps-rubella (MMR) vaccine really be a “low-risk–high-reward” measure in COVID-19? However, I have several concerns about their opinion/hypothesis posed in their article (1).

First, global ecological/epidemiological data might have suggested a correlation between MMR vaccination rate and decreased COVID-19 mortality (1). Some researchers disagreed with this conclusion for the live vaccines including MMR other than BCG (2). Some countries like Iran and Latin American countries, e.g., Chile, Argentina, and Ecuador, maintaining >90% vaccine coverage, which started BCG vaccination in 1985 or even earlier, still have high mortality from COVID-19 (2, 3). Other possible elements such as individual ACE2 receptor and HLA expressions should be kept in mind. Consistently, previous studies have demonstrated HLA-B*4601 expression to be related to a higher risk of developing severe acute respiratory syndrome (SARS) infection and its dire outcome (4).

Second, the presumed trained immunity and/or nonspecific effects (NSE) of live vaccines excluding BCG have not been proven in a study (2, 3, 5). The NSE of the BCG vaccine have also not been well investigated in human beings, and even their clinical relevance is unknown in mouse models. BCG was detected to stimulate a trained immune response to avian influenza virus A (H7N9) in a mouse model, though it was not related to clinical parameters and distinction in survival, or lung inflammation (5). Moreover, if NSE occur against lethal infections and improve host responses against subsequent infections, as mentioned in the article (1), one could expect to see the same effect in influenza mortality. This kind of immunity indeed should not prefer any microbes and should show the same effect against all viruses/pathogens such as in SARS/Middle East respiratory syndrome (MERS) infections.

Third, the authors suggested that one of the explanations for children being resistant to viral infections is their recurrent contact with other live childhood vaccines (1). However, it is hard to associate just MMR with COVID-19, since the other set of live vaccines (BCG, polio, rotavirus, and chickenpox) also are administered at less than 1 year of age. Could this be a cumulative effect?

Fourth, as said, if MMR immunization will be a preventive measure, when should it be administered? How long does the supposed trained immunity caused by MMR continue after vaccination? Earlier research has demonstrated that the NSE of live vaccines such as in BCG on monocytes persist for a couple of months, but certain effects, specifically the augmented capacity of monocytes to release cytokines, slowly diminish afterward (6). As a result, how could the authors relate the incidence/mortality of COVID-19 in adults to a live vaccine administered at 1 year of age?

It is obvious that randomized controlled further clinical investigations are required to find out the real relationship between live vaccines and COVID-19 disease development.
